# Identifying Postural Instability in Children with Cerebral Palsy Using a Predictive Model: A Longitudinal Multicenter Study

**DOI:** 10.3390/diagnostics13122126

**Published:** 2023-06-20

**Authors:** Carlo Marioi Bertoncelli, Domenico Bertoncelli, Sikha S. Bagui, Subhash C. Bagui, Stefania Costantini, Federico Solla

**Affiliations:** 1 Department of Computer Science, Hal Marcus College of Science & Engineering, University of West Florida, Pensacola, FL 32514, USA; domenico.bertoncelli@gmail.com (D.B.); bagui@uwf.edu (S.S.B.); sbagui@uwf.edu (S.C.B.); 2 EEAP H Germain and Department of Pediatric Orthopaedic Surgery, Lenval Foundation, University Pediatric Hospital of Nice, 06000 Nice, France; federico.solla@hpu.lenval.fr; 3 Department of Information Engineering, Computer Science and Mathematics, University of L’Aquila, 67100 L’Aquila, Italy; stefania.costantini@univaq.it

**Keywords:** artificial intelligence in diagnostics, prediction model, machine learning, postural instability, cerebral palsy, truncal tone

## Abstract

Insufficient postural control and trunk instability are serious concerns in children with cerebral palsy (CP). We implemented a predictive model to identify factors associated with postural impairments such as spastic or hypotonic truncal tone (TT) in children with CP. We conducted a longitudinal, double-blinded, multicenter, descriptive study of 102 teenagers with CP with cognitive impairment and severe motor disorders with and without truncal tone impairments treated in two specialized hospitals (60 inpatients and 42 outpatients; 60 males, mean age 16.5 ± 1.2 years, range 12 to 18 yrs). Clinical and functional data were collected between 2006 and 2021. TT-PredictMed, a multiple logistic regression prediction model, was developed to identify factors associated with hypotonic or spastic TT following the guidelines of “Transparent Reporting of a multivariable prediction model for Individual Prognosis or Diagnosis”. Predictors of hypotonic TT were hip dysplasia (*p* = 0.01), type of etiology (postnatal > perinatal > prenatal causes; *p* = 0.05), male gender, and poor manual (*p* = 0.01) and gross motor function (*p* = 0.05). Predictors of spastic TT were neuromuscular scoliosis (*p* = 0.03), type of etiology (prenatal > perinatal > postnatal causes; *p* < 0.001), spasticity (quadri/triplegia > diplegia > hemiplegia; *p* = 0.05), presence of dystonia (*p* = 0.001), and epilepsy (refractory > controlled, *p* = 0.009). The predictive model’s average accuracy, sensitivity, and specificity reached 82%. The model’s accuracy aligns with recent studies on applying machine learning models in the clinical field.

## 1. Introduction

Cerebral palsy (CP) is a set of nonprogressive postural and motor control disorders caused by injury in the early stages of brain development [1]. Postural control involves the control of the body’s position in space, orientation, and stability [2,3]. This stabilization is necessary for free and selective movement of the head and extremities. Trunk control provides an initial framework for postural control, controls selective trunk movement stabilization, and ensures the development of gross motor skills. It forms the basis for developing goal-directed activities essential for independent living at home or in the community. A stable trunk also aids in developing the child’s cognitive, social, and communication skills, promoting the child’s orientation to himself or herself and the environment [2,3].

Children with CP often show deficits in the development of postural control. In the motor dysfunction of children with CP, postural control problems, including those of the trunk, play a significant role. The exact nature of the deficits is only known to a limited extent. There are many studies on postural control in children with CP in the literature, but most focus on standing [4,5]. The infants with spastic hemiplegia showed direction-specific adjustments from 15 months onwards. Unlike typically developing infants, they did not develop the ability to modulate EMG amplitude to the velocity of the reaching arm or to the initial pelvis position in the age period till 18 months [5].

In terms of muscle recruitment strategies, postural control can be considered to consist of two levels. The first or basic level is direction specificity, meaning the forward body sway is counteracted by the activation of dorsal muscles. The second level consists of fine-tuning the postural adjustment, for example, in the number of muscles activated, the recruitment order, and the use of anticipatory adjustments. In infants at high risk of CP, no increase in direction specificity was observed between 6 and 18 months [6].

For limb movements, the trunk is an indispensable element. It has an inter-functional connection with other body parts and the nervous system. Children with hypertonic/spastic or hypotonic trunk tone (TT) have a reduced ability to maintain an upright position. A strong preference for a top-down recruitment of the postural muscles in children with CP is found during perturbation experiments in sitting and standing positions [5]. Children with CP show a high amount of antagonistic co-activation during perturbation experiments in sitting and standing positions. In the sitting position, co-activation is especially high when perturbations induce a backward body sway. Conversely, little antagonistic co-activation is found when perturbations induce a forward sway of the body [5,6]. The major postural dysfunction of children with CP is the substantially reduced capacity to modulate the degree of postural muscle contraction to the specifics of the situation. For instance, children with CP have difficulties using information from the initial body configuration to adapt to postural activity [5]. This results in reduced postural control and delayed achievement of gross motor milestones [5,6,7].

In many children with CP, trunk stabilization is an essential biomechanical component influencing head stability, visual field orientation, and hand manipulation. Consequently, a more effective solution would be to provide intermediate trunk stabilization levels rather than full support. However, the optimal level of trunk support for a child with moderate-to-severe CP remains unknown [8,9,10].

Machine learning (ML) is a contemporary application of artificial intelligence (AI) for studying complex data, using algorithms to detect patterns in data that are not discernible to humans. Regression and logistic regression are among the earliest supervised ML algorithms used to build predictive models in healthcare [11].

ML algorithms are considered supervised if the output classes are known (e.g., hypotonic/spastic TT). Thus, supervised machine learning prediction models enable the detection of associated factors of TT instability, such as hypotonic or spastic. Prediction models can help assess the likelihood of a disease (e.g., hypotonic/spastic TT) [11,12,13].

In supervised ML algorithms, the results of some inputs (training examples) are uniquely defined. The program learns a function (e.g., logistic regression) through the training examples to predict incoming patients with unspecified health statuses.

A supervised learning algorithm involves the following steps [14]:

1. Data collection on a training set and a test set;
2. Choosing a function to be learned (e.g., a logistic regression);
3. Running the learning algorithm on the training sets;
4. Optimizing the parameters of the function (e.g., regression coefficients);
5. Checking the accuracy of the learned function on the test set.

A vector of information that includes multiple variables generally characterizes each patient. The number of variables must contain enough information to predict the outcome but should not be too numerous. Following the steps described earlier, supervised ML logistic regression is applied in the supervised ML algorithm. Logistic regression estimates the probability that a patient will have a certain outcome (e.g., hypotonic/spastic TT).

In previously published studies, a supervised ML model called “PredictMed” [15,16] was developed [17] and validated [18] to predict hip dysplasia and neuromuscular scoliosis. This algorithm was also used to identify factors associated with intellectual disabilities, autism spectrum disorders, and gastrostomy placement in CP patients [19,20,21,22].

Although there are many publications on postural instability, we have not found specific studies on the type of truncal tone impairments (hypotonic and spastic) in subjects with CP. Our study aims to identify the factors associated with hypotonic/spastic truncal tone in adolescents with CP using TT-PredictMed, a supervised machine learning model.

## 2. Materials and Methods

### 2.1. Study Design

A longitudinal, double-blind, descriptive, multicenter study was conducted between June 2007 and June 2021. While data analysis began in June 2019 and continued for 24 months, data collection and evaluation for model implementation were performed in the last 6 months of 2022.

### 2.2. Subjects

**Figure 1:**
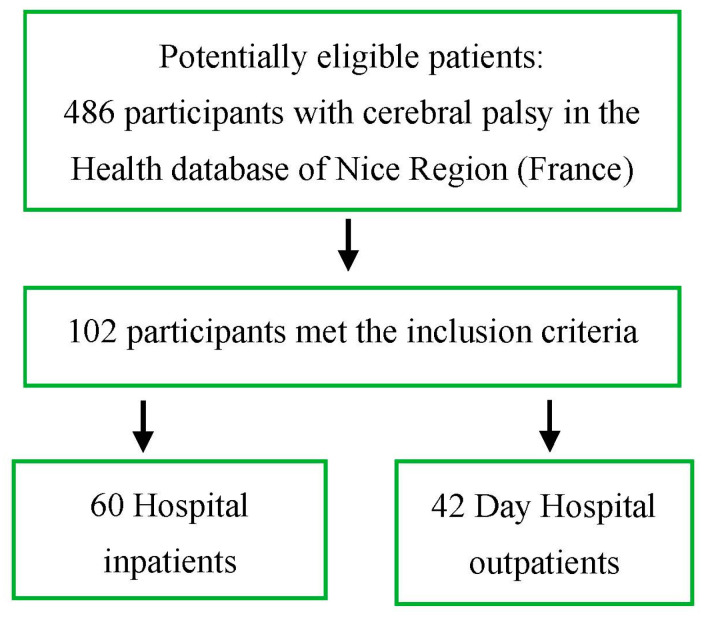
Flow diagram of study participants for analysis.

We have collected data from two cohorts: long-term patients living in institutional care and others treated in day hospitals. Among 486 children with CP in the Nice region (France), 102 subjects, 60 from Lenval University Children’s Hospital and 42 from Nice Day Hospital (age 16.5 ± 1.2 years, 60 males, 42 females) (Figure 1), met the following inclusion criteria: age 12 to 18 years at last follow-up, spastic, dystonic, with mixed spastic/dystonic or hypotonic CP, classified according to the European Cerebral Palsy Surveillance System [23], and had at least three years of follow-up (6.4 ± 1.2 years, range 3–12). Spinal cord neuropathology and progressive encephalopathy were the exclusion criteria. There were no missing data.

### 2.3. Measurements

The senior author collected the medical records. He also assembled medical notes prepared by a multidisciplinary team, including orthopedic surgeons, pediatric neurologists, pediatricians, epidemiologists, and physical therapists (Table 1). The notes were also coded narrative notes compiled in the “TT-PredictMed” database [15,16]. Implementation of the model followed the guidelines of the statement “Transparent Reporting of a multivariable prediction model for Individual Prognosis or Diagnosis” (TRIPOD) [13].

The etiology of CP has been classified as prenatal (brain malformation, genetic, vascular, or infectious), perinatal (ischemic, anoxic, or infectious), or postnatal (infectious, head trauma, postnatal anoxic/ischemic injury, or epilepsy) [15,16]. The Gross Motor Function Classification System (GMFCS) and the Manual Ability Classification System (MACS) [17,18] were used to assess motor function. The Eating and Drinking Ability Classification System for Individuals with Cerebral Palsy (EDACS) was applied to evaluate the eating and drinking ability [15,20]. GMFCS, EDACS, and MACS provide a 5-point grading system, with higher scores indicating worsening motor functioning.

### 2.4. Assessment of Trunk Postural Control

We assessed trunk muscle tone with the Trunk Control Measurement Scale (TCMS) [24,25,26] and the Trunk Impairment Scale (TIS) [27,28], which involved dynamic and static balance scores for the sitting position. The subdivision of muscle tone into hypotonic, spastic, or normal was stated to measure trunk control in adults after stroke. It was accomplished by assessing static and dynamic seated balance (DSB) and trunk coordination in a sitting position. The total score calculation consists of the sum of the scores of the three subscales, ranging from 0 (lowest performance) to 23 (best performance).

**Figure 2:**
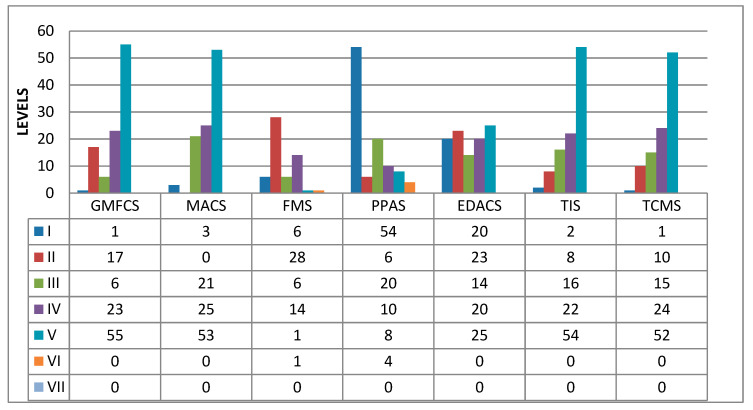
Distribution of patients according to the Manual Ability Classification System (MACS), Gross Motor Function Classification System (GMFCS), Posture and Postural Ability Scale (PPAS), Functional Mobility Scale (FMS), Eating and Drinking Ability Classification System (EDACS), Trunk Control Measurement Scale (TCMS), and Trunk Impairment Scale (TIS).

The TCMS was developed from the TIS and is an expanded version. The TCMS contains more information on selective trunk control and dynamic reaching. The total score ranges from 0 to 58. The total score is the sum of the scores of the three subscales: static seated balance, dynamic seated balance–selective trunk control (DSB-S), and dynamic seated balance–recollection (DSB-R). In total, it contains 15 items. The items are assigned on a two-, three-, or four-point scale. The maximum subscale scores are 20, 28, and 10, respectively. Its reliability and validity have been demonstrated in children and adolescents with CP aged 5–19 years [4,28,29,30] (Figure 2).

The children were examined in a quiet environment during their private training practice. The same physical therapist with clinical experience in children with CP performed all assessments. TIS was performed while the child sat on a bench, with hands and forearms resting on the thighs, without back support. TCMS was performed while the child sat on a bench without arm, back, or foot support. Each child began each exercise with the trunk in the most upright position and had to maintain this position as much as possible while performing the exercises [4].

The Functional Mobility Scale (FMS) [31], the Lower Extremity Functional Scale (LEFS) [32], and the Posture and Postural Ability Scale (PPAS) [33] were used to assess functional ability. FMS measures functional mobility and walking ability on a 6-level grading system. Higher scores indicate better motor functioning. LEFS assesses lower limb function on an 80-point scale, with higher scores indicating better functional status. PPAS assesses sitting posture on a 7-level grading system, with higher scores indicating better postural control (Figure 1).

The presence of a Cobb angle defines scoliosis > 10° on the spine radiograph and is considered “severe” when the Cobb angle is >40° [17,18] (Table 1). Neurological status was determined by the presence of hypertonia or hypotonia in the upper or lower extremities, anatomy of the spastic disorder (hemiplegia, diplegia, tri/quadriplegia), severity of epilepsy, and presence of dystonia.

Spasticity has been assessed with the modified Ashworth scale of Bohannon and Smith and the modified Tardieu scale [17,18]. Epilepsy severity has been determined by pediatric neurologists and considered “well controlled” or “intractable” [34], according to the International League Against Epilepsy. The International League Against Epilepsy considers epilepsy intractable/continuous seizures when treatment attempts have been made with at least two antiepileptic drugs [35,36] (Table 1).

Hip dysplasia was assessed according to the Perkins line. Zero was assigned if the lateral margin of the femoral head was medial to the Perkins line and the migration percentage (MP) was negative. The migration percentage (MP) was evaluated as 100% when the entire femoral head was lateral to the Perkins line. Based on the migration percentage, hips were identified as normal (MP less than 33%), subluxated (MP = 33 up to 89%), or dislocated (MP ≥ 90%) [37].

**Figure 3:**
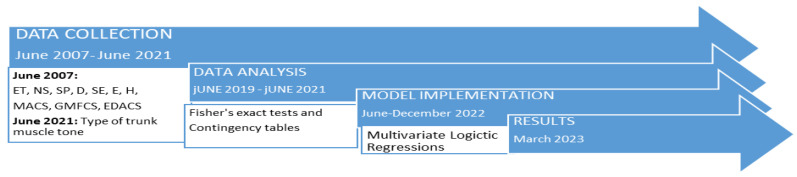
Chronology of the study measurements and prediction model implementation. Type of etiology (ET), sex (SE), presence of dystonia (D), spasticity (SP), epilepsy (E), neuromuscular scoliosis (NS), hip dysplasia (H), Gross Motor Function Classification System (GMFCS), Manual Ability Classification System (MACS), Eating and Drinking Ability Classification System for Individuals with Cerebral Palsy (EDACS).

The type of etiology (ET), sex (SE), presence of dystonia (D), spasticity (SP), epilepsy (E), neuromuscular scoliosis (NS), hip dysplasia (H), MACS, EDACS, and GMFCS were assessed at the first check; the type of trunk muscle tone (hypotonic, spastic, or normal) was assessed at the last check (Figure 3).

### 2.5. Statistical Analysis

Fisher’s exact tests were used to calculate the confidence intervals and distribution frequencies of the risk factors of hypotonic/spastic TT, and contingency tables were created [38]. Using OpenEpi 3.01, a web-based epidemiological calculator, and MedCalc^®^ 20.123 statistical software, we derived 95% confidence intervals, odds ratios, and z-statistics (Table 2) [15]. Common thresholds (*p*-value < 0.2) [38] were used to choose independent input variables in a customized multiple logistic regression model. We have reduced the number of independent variables using the feature reduction mode to eliminate redundant variables (e.g., eliminating predictors having interactions) maximizing the predictive performance by eliminating redundancy.

We aimed to predict each patient’s probability of having hypotonic or spastic TT, using the glm() function of the open source software R 4.2.2 [15].

Dependent variables were the presence of hypotonic, spastic, or normal TT. Independent variables were the type of spasticity (SP), etiology (ET), presence of epilepsy (E), dystonia (D), hip dysplasia (H), neuromuscular scoliosis (NS), gender (SE), MACS, GMFCS, and EDACS.

We divided the patient data into a “training set” and a “test set” according to the statistical learning theory described by Vapnik [14,15]. The logistic regression algorithm was trained on the “training set” of 80 patients (later excluded from the test set) based on the values of the best-selected independent variables, ET, SP, D, E, NS, H, SE, GMFCS, MACS, and EDACS (Table 3), to predict the probability of a new patient having hypotonic or spastic TT (belonging to the “test set” of 22 patients). We applied cross-validation by randomly generating 20 different pairs of training and test sets, i.e., the compositions of the training and test sets were randomly changed in 20 rounds of cross-validation to minimize the dependence on the compositions of the training and test sets. We then calculated the accuracy, sensitivity, and specificity of the predictions for each pair and determined the mean [15].

As usual, the specificity, sensitivity, and accuracy of the predictions were described in terms of true positive (TP), true negative (TN), false negative (FN), and false positive (FP). Specificity was considered as the percentage of actual negatives that were correctly identified as such. Sensitivity was identified as the percentage of actual positives identified as such. Accuracy was described as the precision of the measurement system in relation to reproducibility and repeatability and was calculated as (TP + TN)/(TP + TN + FP + FN) [15].

Through the R predict function glm(), we tested the model’s predictive ability on a new test set. For each patient in the test set, it produced a probability (Prob) of having hypotonic or hypertonic TT in the form of Prob [TT = yes|glm (ET + NS + SP + D + SE + E+ H + MACS + GMFCS + EDACS)], where 0 < Prob < 1. The threshold was established for the decision limit. If Prob [TT = yes|glm (ET + NS + SP + D+ SE + E + H+ MACS + GMFCS + EDACS)] > threshold, the presence of hypotonic or spastic TT was predicted. 

Thresholds from 0.1 to 0.8 were tested, and the results’ specificity, sensitivity, and accuracy were compared [15]. For example, choosing 0.5 means that if the probability of developing hypotonic or spastic TT predicted by the logistic regression model for a given subject is >0.5, then that subject is classified as a potential developer of (or affected by) hypotonic or spastic TT. Suppose the patient (belonging to the test set) is affected by hypotonic or spastic TT. In such a case, he or she is classified as a TP (true positive) instead of FP (false positive). Similarly, patients were identified as TN (true negative) or FN (false negative) [15]. Once this was accomplished for each patient in the “test set”, we compared the predictions with the patient’s known state (e.g., whether or not they had hypotonic or spastic TT) to determine the accuracy, specificity, and sensitivity of the predictive logistic regression.

## 3. Results

Table 1 shows the clinical presentation according to the presence of hypotonic or spastic TT. Functional, postural, motor, and eating skills are summarized in Figure 1. Regarding motor function, 76% (*n* = 78) had MACS ≥ 4 (mean 4.3 ± SD 1.4) and GMFCS ≥ 4 (mean 4.3 ± 1.2). Concerning trunk movement skills, 59% (*n* = 60) had only static sitting balance, 37% (*n* = 38) possessed dynamic sitting balance, 4% (*n* = 4) had good postural balance, and none achieved the maximum TIS score of 23 points. TCMS showed comparable results (Figure 1). Trunk muscle tone problems were detected in 53% of cases (*n* = 54, 5 hypotonic, 49 spastic). Half of the subjects were unable to walk for functional mobility, 47% (*n* = 48) were able to walk with aids, and only 2% (*n* = 2) could walk self-sufficiently. Regarding lower limb function, 49% (*n* = 48) had considerable difficulty, 35% (*n* = 36) had severe difficulty, 18% (*n* = 18) had modest difficulty, and none had mild or no difficulty. Regarding postural ability, 59% (*n* = 60) could not sit or needed support to maintain an aligned sitting position, and 29% (*n* = 30) were able to sit and could move. In comparison, 12% (*n* = 12) could shift their weight laterally and leave the sitting position.

The neurological status of epilepsy, dystonia, spasticity, neuromuscular scoliosis, and hip dysplasia is shown on Table 1.

Regarding the hip range of motion, uni- or bilateral internal rotation was >40° in 27% (*n* = 27) of patients, and 56% (*n* = 57) showed reduced hip abduction <20°. Radiologic hip morphology indicated that 61% (*n* = 62) had normal or near-normal hips, 11% (*n* = 11) had dysplastic hips, 6% (*n* = 6) had dysplasia with mild subluxation, 4% (*n* = 4) had moderate to severe subluxation, and 4% (*n* = 4) had dislocated hips (Table 1).

In the univariate analysis, the following variables were associated with truncal tone disorders (hypotonic/spastic): the presence of hip dysplasia (*p* = <0.0001), severe scoliosis (*p* = 0.0024), epilepsy (*p* = 0.0043), dystonia (*p* = 0.0466), and manual, gross motor, and eating disorders (*p* = <0.0001) (Table 2).

Multivariate regression reported that the factors most associated with hypotonic TT were the following:

- Hip dysplasia: *p* = 0.01, OR = 23.18;
- Manual disability: *p* = 0.01, OR = 16.98;
- Male gender: *p* = 0.03, OR = 18.5;
- Gross motor function: *p* = 0.05, OR = 6.17;
- Type of etiology: postnatal > perinatal > prenatal causes, *p* = 0.05, odds ratio (OR) = 0.15.

Factors associated with spastic TT were the following:

- Presence of dystonia: *p* = 0.001, OR = Infinite;
- Type of etiology: prenatal > perinatal > postnatal causes, *p* < 0.001, OR = Infinite;
- Type of epilepsy: controlled > refractory, *p* = 0.009, OR = Infinite;
- Neuromuscular scoliosis: *p* = 0.03, OR = Infinite;
- Type of spasticity: quadri/triplegia > diplegia > hemiplegia, *p* = 0.05, OR = Infinite.

The best multivariate model scored 84% accuracy, 71% sensitivity, and 90% specificity (Table 3).

The “Pr(>|z|)” column at the far right of Table 3 indicates the significant strength of the respective parameters in terms of the *p*-value as a predictor of truncal tone disorder. This means that the significance of SE, H, GMFCS, MACS, and ET in predicting hypotonic truncal tone and H, NS, ET, SP, D, and E in predicting hypertonic truncal tone is very probable, with a *p*-value < 0.05.

## 4. Discussion

Although many publications about impaired postural control exist, we have not found specific comparative studies on the hypotonic and spastic trunk muscles in subjects with CP using a predictive model. Hence, we developed, tested, and applied a machine-learning model to identify factors associated with hypotonic or spastic TT in teenagers with CP. 

Previous studies have identified high levels of GMFCS [4,9,25,26,30] and spasticity [4,39,40] associated with postural disorders. The degree of trunk impairment depends on the severity and topography of motor impairment in CP [4]. The present study confirms these results. More precisely, we found that high levels of GMFCS and MACS have a strong association with hypotonic TT. Children with GMFCS V have limited trunk control but respond to support similar to young, typically developing infants, suggesting delayed postural control. Response to external support for children with GMFCS IV suggests that a unique strategy for trunk control not observed in typical infants [9]. 

For CP children with trunk hypotonia, the difficulty in achieving upper limb ability and gross motor skills entails significant clinical implications. Thus, it is recommended that, in such children, trunk control ability be assessed when evaluating upper limbs and gross motor functions [41].

In line with the literature [4,39,40], we establish that the risk of spastic TT varies according to CP subtype: 5% for spastic hemiplegia, 5% for diplegia, 36% for tri/quadriplegia, and 28% for dystonia. This hypertonicity may underlie functional impairments of posture and locomotion. The axial and appendicular tones seem to be controlled by separate neural circuits [42].

The interconnection between trunk control, leg muscle strength, and selectivity for gait capacity in children with CP has been shown [30]. It indicates the significance of postural impairments in gait assessment and, potentially, rehabilitation. Lower extremity muscular strength, selectivity, and trunk control explain a similar amount of gait capacity variance, which is higher than that explained by lower extremity spasticity. Lower extremity muscle strength and selectivity is strongly correlated with trunk control. Therefore, we cautiously suggest that combined trunk control and lower extremity training might be promising for improving gait capacity in children with CP (GMFCS level I–III), which needs to be tested in future intervention studies [30].

In addition to well-known factors, we identified the following new predicting variables of hypotonic and spastic TT: type of etiology, male gender, presence of dystonia, and epilepsy. However, neuromuscular scoliosis and hip dysplasia could be associated factors rather than etiologic.

We underlined the link between trunk muscle tone disorder and type of etiology: antenatal and perinatal rather than postnatal causes seem to be risk factors of spastic TT. The inverse association has been found for hypotonic TT. Because the prediction model has a low odds ratio and there are no studies on this, future research is needed to confirm this finding.

On the orthopedic side, previous studies stated that teenagers with CP, neuromuscular scoliosis [18], and hip dysplasia [19] were four times more likely to develop TT instability. The present study specifies that hip dysplasia is associated with hypotonic and spastic TT. This has a strong odds ratio, while neuromuscular scoliosis seems to be associated with less incisiveness and mainly with spastic TT.

Another novelty concerns male CP subjects, particularly at risk of hypotonic TT compared to female participants. Given the small number of study participants and the lack of the relevant literature, we are very cautious in interpreting this link. However, we intend to explore this aspect with a larger cohort of participants in the future. 

On the neurologic side, we found that CP subjects with dystonia and epilepsy (refractory > controlled) are more at risk of spastic TT than the control group. 

Epilepsy and dystonia could result from the comorbidity of a deficient nervous system with musculoskeletal disorders; encephalopathies causing epilepsy commonly affect motor control. This could cause truncal or postural tone disorders. The association between epilepsy and truncal tone disorders can be explained by early cortical injury [15,43]. The literature is scanty on this, probably due to the difficulty of combining epilepsy patient data with orthopedic data.

Identifying the type of postural instability (spastic versus hypotonic) in CP as early as possible should allow us to anticipate and personalize rehabilitation treatments. Infants with truncal hypertonicity at 3 months have significantly lower Bayley motor and mental scores at 18 months than infants with normal truncal tone. However, infants with lower extremity hypertonicity at 3 months are no different developmentally at 18 months from infants with normal tone. Infants with truncal or lower extremity hypotonicity fared the worst developmentally. Therefore, there is a high incidence of abnormal muscle tone in premature infants up to 18 months of age and early truncal tone abnormalities are associated with a worse developmental outcome [43]. This deserves particular attention in assessing and treating tone disorders in infants with (or at high risk of) CP. 

The main clinical implications of our findings include:

- Using a prediction model to identify risks and factors associated with the type of TT (hypotonic/spastic) in children with moderate–severe CP. This will help clinicians anticipate and focus on rehabilitation strategies and provide guidance toward implementing optimal levels of trunk assistance during rehabilitation [25].
- Trunk control should be one of the principal focus areas in testing and intervention in this population. So far, the assessment of truncal muscle tone in CP subjects has only focused on the correlation between trunk muscle deficit and functional abilities. Results from our study indicate that further characteristics related to the trunk’s hypotonia or spasticity should be considered. These factors should be evaluated when planning rehabilitation programs for CP children with trunk tone disorder.
- Early identification of those at risk for TT instability would help the child achieve independence. Adolescents with the highest independence in motor activity are further interested in conducting activities. The interest in performing activities is associated with maximum independence in motor performance, personal values, and social environment support. Thus, physical rehabilitation for adolescents with CP is recommended, as it can improve their motivation [44].

Children and families who face the daily consequences of postural control deficits have long demanded more attention from clinicians and researchers. Therefore, it is a surprise that so little consideration has been given to the critical role trunk control can play in the motor deficits these children manifest. Improvements over the past decade have shown that trunk control can be investigated in a clinically meaningful way with greater accuracy [41].

We hope this study will be helpful to in supporting clinical decision making for improving functional skills in CP children with trunk postural control problems. This can be achieved by focusing more closely on the type of TT deficit. 

**Figure 4:**
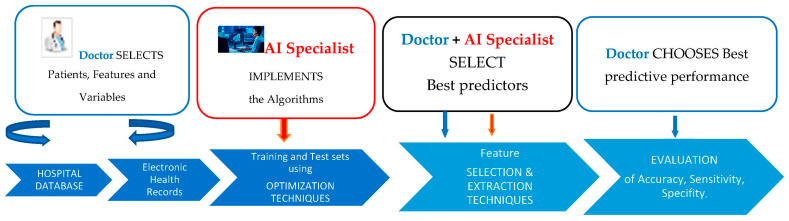
PredictMed: Roles and Tasks for the Implementation of the Prediction Model.

This mode of clinical reasoning may be critical in more precisely targeting an intervention to increase functional skills in subjects with CP [25]. The complex interplay of medical and machine learning skills provides new perspectives for personalized patient care (Figure 4).

### Limitations

Limitations of this study include the relatively small number of patients and the retrospective analysis. It is essential to stabilize the predictive performance of the algorithm through a significant increase in the number of patients while keeping the number of independent variables unchanged (<15). Although we limited the number of patients (hundreds) for this study, we intend to implement and refine the model with a more significant number of patients (thousands) in the future. This will help us test and optimize the predictive performance of the model. The limited number of patients also results in potential overfitting of the model, which will be solved by studying a large number of patients [16,19].

## Figures and Tables

**Table 1:** Clinical presentation according to the type of truncal tone.

	Type of Truncal Tone			
Patient Profile	Normal (%)	Hypotonic (%)	Spastic (%)	Total (%)
Patients *n*. (%)	48 (47)	49 (48)	5 (5)	102 (100)
Male	27 (45)	31 (52)	2 (3)	60 (100)
Female	21 (50)	18 (43)	3 (7)	42 (100)
Average age (mean, SD)	16.7 (1.87)	16.8 (1.87)	16.4 (1.87)	16.6 (1.87)
Spasticity *n*. (%)	29 (38)	42 (55)	5 (7)	76 (100)
Hemiplegia	7 (58)	5 (42)	0 (0)	12 (100)
Diplegia/Paraplegia	10 (67)	5 (33)	0 (0)	15 (100)
Tri/quadriplegia	12 (24)	32 (65)	5 (11)	49 (100)
Dystonia *n*. (%)	3 (21)	9 (75)	2 (4)	14 (100)
Well-controlled Epilepsy, *n*. (%)	22 (44)	26 (52)	2 (4)	50 (100)
Intractable Epilepsy	4 (18)	17 (77)	1 (5)	22 (100)
No epilepsy *n*. (%)	21 (70)	7 (23)	2 (7)	30 (100)
Severe Scoliosis (%)	11 (17)	51 (78)	3 (5)	65 (100)
Hip Dysplasia (%)	6 (9)	55 (86)	3 (5)	64 (100)
Antenatal Causes	33 (52)	30 (47)	1 (1)	64 (100)
Perinatal Causes	10 (38)	12 (46)	4 (6)	26 (100)
Postnatal Causes	5 (42)	7 (58)	0 (0)	12 (100)

**Table 2:** Contingency table comparing the groups (with and without truncal tone disorders) using contingency tables and Fisher’s exact test.

Independent Variables		Truncal Tone Disorders		Fisher’s Exact Test *p* Value	Odds Ratio Estimate	95% Confidence Intervals
		Yes	No			
Presence of Hip Dysplasia	Yes	22	42	<0.0001	0.098	0.03–0.27
	No	32	6			
GMFCS ≥ 4/5	Yes	52	26	<0.0001	22.00	4.80–100
	No	2	22			
MACS ≥ 4/5	Yes	52	26	<0.0001	22.00	4.80–100
	No	2	22			
EDACS ≥ 4/5	Yes	37	8	<0.0001	10.88	4.20–28.1
	No	17	40			
Severe Scoliosis	Yes	26	39	0.0024	4.0238	1.63–9.89
	No	28	9			
Presence of Spasticity	Yes	47	29	0.0029	4.399	1.64–11.7
	No	7	19			
Presence of Epilepsy	Yes	45	27	0.0043	3.888	1.55–9.71
	No	9	21			
Presence of Dystonia	Yes	11	3	0.0466	3.837	1.00–14.7
	No	43	45			

**Table 3:** List of the multinomial logistic regression coefficients (independent variables) associated with the presence of hypotonic and spastic truncal tone. a. Logistic Regression for Hypotonic Truncal Tone: SE (male gender) and increasing (positive values) H, GMFCS, and MACS, as well as decreasing (negative values) ET (postnatal > perinatal > prenatal causes) are predictive factors of hypotonic truncal tone (in the “Estimate” column). As an example, this means that for every unit increase in H, the log odds = ln(p/1 − p) increase 3.143 times (where p = probability to develop hypotonic truncal tone), while for every unit decrease in ET, the log odds = ln(p/1 − p) decrease −1.851 times for ET. b. Logistic Regression for Spastic Truncal Tone: The increase in H, ET (prenatal > perinatal > postnatal causes), SP, and D (positive values), as well as the decrease (negative values) in NS and E, are predictive factors of hypertonic truncal tone (in the “Estimate” column).

Independent Variables	Logistic Regression				
	Odds Ratio Estimate		Standard Error	Z Ratio	Prob (>|z|)
	Logarithm	Linear			
Intercept	−21.11	6.792e	5.9411	−3.5544	0.0003
Hip Dysplasia (H)	3.143	23.18	1.2301	2.5557	0.0105
Scoliosis (NS)	−0.801	0.448	1.0585	−0.7576	0.4487
Type of Etiology (ET)	−1.851	0.157	0.9797	−1.8894	0.0500
Type of Spasticity (SP)	0.829	2.292	0.4655	1.7819	0.0747
Dystonia (D)	6.729	836.7	4.4937	1.4975	0.1342
Male gender (SE)	2.916	18.48	1.3443	2.1698	0.0300
GMFCS score	1.820	6.176	0.9459	1.9249	0.0500
MACS score (M)	2.832	16.98	1.1162	2.5374	0.0111
Type of Epilepsy (E)	1.094	2.987	0.9700	1.1281	0.2592
EDACS score	−0.457	0.632	0.4773	−0.9592	0.3374
Intercept	−243.64	1.543e	8.9175	−27.321	0.0001
Hip Dysplasia (H)	59.010	4.243e	23.289	2.5338	0.0112
Scoliosis (NS)	−38.634	1.671e	18.426	−2.0967	0.0365
Type of Etiology (ET)	85.402	1.229e	8.2450	10.358	0.0001
Type of Spasticity (SP)	51.871	3.367e	26.752	1.9389	0.0500
Dystonia (D)	80.736	1.156e	26.068	3.0971	0.0019
Gender (SE)	−41.882	6.482e	30.973	−1.3522	0.1763
GMFCS score	−1.7571	0.172	37.175	−0.0473	0.9623
MACS score (M)	−22.928	1.111e	76.845	−0.2984	0.7654
Type of Epilepsy (E)	−24.261	2.910e	9.3918	−2.5832	0.0097
EDACS score	4.5932	98.81	24.740	0.1857	0.8527

## Data Availability

Not applicable.
